# P-1501. Effectiveness of 2024–2025 seasonal influenza vaccines against influenza–associated hospitalizations among adults — VISION Network

**DOI:** 10.1093/ofid/ofaf695.1685

**Published:** 2026-01-11

**Authors:** Jennifer DeCuir, Emily L Reeves, Zachary Weber, Duck-Hye Yang, Stephanie Irving, Sara Y Tartof, Nicola P Klein, Shaun J Grannis, Toan Ong, Sarah W Ball, Malini B DeSilva, Kristin K Dascomb, Allison L Naleway, Padma Kppolu, S Bianca Salas, Lina S Sy, Bruno Lewin, Richard Contreras, Ousseny Zerbo, John R Hansen, Lawrence Block, Karen B Jacobson, Brian E Dixon, Colin Rogerson, Thomas Duszynski, William F Fadel, Michelle Barron, David Mayer, Catia Chavez, Adam Yates, Lindsey Kirshner, Charlene E McEvoy, Omobosola Akinsete, Inih Essien, Tamara Sheffield, Daniel Bride, Julie Arndorfer, Joshua Van Otterloo, Karthik Natarajan, Caitlin S Ray, Amanda B Payne, Shikha Garg

**Affiliations:** Centers for Disease Control and Prevention, Atlanta, GA; Centers for Disease Control and Prevention, Atlanta, GA; Westat, Rockville, Maryland; Westat, Rockville, Maryland; Kaiser Permanente Center for Health Research, Portland, Oregon; Kaiser Permanente Southern California, Pasedena, CA; Division of Research Kaiser Permanente Vaccine Study Center, Oakland, California; Indiana University, Indianapolis, Indiana; University of Colorado Anschutz Medical Campus, Centennial, Colorado; Westat, Rockville, Maryland; HealthPartners Institute, Bloomington, Minnesota; Intermountain Healthcare, Murray, Utah; Kaiser Permanente Center for Health Research, Portland, Oregon; Kaiser Permanente Center for Health Research, Portland, Oregon; Kaiser Permanente Southern California, Pasedena, CA; Kaiser Permanente Southern California, Pasedena, CA; Kaiser Permanente Department of Research and Evaluation, Pasadena, CA; Kaiser Permanente Southern California Department of Research & Evaluation, Pasadena, California; Division of Research Kaiser Permanente Vaccine Study Center, Oakland, California; Division of Research Kaiser Permanente Vaccine Study Center, Oakland, California; Kaiser Permanente Northern California, Oakland, California; Kaiser Permanente Vaccine Study Center, Oakland, California; Regenstrief Institute, Indianapolis, Indiana; Regenstrief Institute, Indianapolis, Indiana; Regenstrief Institute, Indianapolis, Indiana; Regenstrief Institute, Indianapolis, Indiana; University of Colorado, Aurora, Colorado; University of Colorado Anschutz Medical Campus, Centennial, Colorado; University of Colorado, Aurora, Colorado; Westat, Rockville, Maryland; Westat, Rockville, Maryland; HealthPartners Institute, Bloomington, Minnesota; HealthPartners Institute, Bloomington, Minnesota; HealthPartners Institute, Bloomington, Minnesota; IntermountainHealth, Salt Lake City, Utah; Intermountain Healthcare, Murray, Utah; Intermountain Healthcare, Murray, Utah; IntermountainHealth, Salt Lake City, Utah; Columbia University, New York, New York; Centers for Disease Control and Prevention, Atlanta, GA; CDC, Atlanta, Georgia; Centers for Disease Control and Prevention, Atlanta, GA

## Abstract

**Background:**

CDC recommends annual influenza vaccination for all persons aged ≥ 6 months. We estimated 2024–2025 seasonal influenza vaccine effectiveness (VE) against influenza–associated hospitalizations among adults.

**Figure 1.**
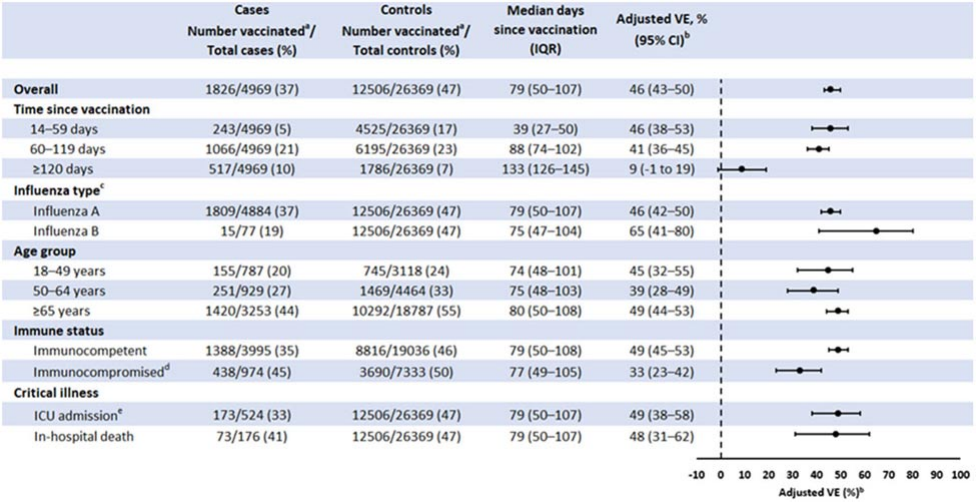
2024–2025 seasonal influenza vaccine effectiveness against influenza–associated hospitalizations among adults aged ≥ 18 years — VISION Network, October 2024–March 2025 Abbreviations: CI = Confidence interval; ICU = Intensive care unit; IQR = Interquartile range; VE = vaccine effectiveness. a) Patients were considered vaccinated if they received ≥1 2024–2025 influenza vaccine dose ≥14 days before the index date, defined as the earlier date of the most recent influenza test and the hospital admission date. b) VE was estimated using multivariable logistic regression models comparing the odds of receipt of ≥1 2024–2025 influenza vaccine dose versus no dose among cases and controls. Models were adjusted for age, sex, race and ethnicity, calendar day, and healthcare system. Age and calendar day were treated as natural cubic splines with 4 degrees of freedom. c) Influenza A and B coinfections were excluded from influenza A and B case counts and from VE estimates against influenza A and B. d) Patients were considered immunocompromised if they had ≥1 ICD-10 discharge diagnosis code for any of the following conditions: hematologic malignancy, solid malignancy, bone marrow transplant, solid organ transplant, rheumatologic/inflammatory disorder, other intrinsic immunodeficiency condition, or HIV/AIDS. e) To estimate VE against ICU admission, cases were restricted to encounters with ICU admission and no in-hospital death.

**Methods:**

Data from the VISION Network were used to estimate influenza VE using a test-negative, case-control design. The analysis included hospitalizations among adults aged ≥ 18 years with ≥ 1 acute respiratory illness (ARI)–associated ICD-10 discharge diagnosis code from October 1, 2024–March 7, 2025 in six US healthcare systems. Cases were ARI hospitalizations with a positive molecular influenza test within 10 days before to 72 hours after the admission date. Controls were ARI hospitalizations with a negative molecular influenza test during the same interval. VE was estimated using multivariable logistic regression comparing the odds of receipt of ≥ 1 2024–2025 influenza vaccine dose versus no dose among cases and controls. VE models were adjusted for age, sex, race and ethnicity, calendar day, and healthcare system.

**Results:**

A total of 31,338 ARI hospitalizations met inclusion criteria, including 4,969 cases and 26,369 controls (Figure). Overall VE against influenza–associated hospitalizations was 46% (95% CI=43–50%) with a median time since vaccination of 79 days (IQR=50–107). When stratified by time since vaccination, VE was 46% (95% CI=38–53%) at 14–59 days, 41% (95% CI=36–45%) at 60–119 days, and 9% (95% CI=-1 to 19%) at ≥ 120 days. VE was 46% (95% CI=42–50%) against influenza A and 65% (95% CI=41–80%) against influenza B. Among immunocompetent and immunocompromised adults, VE was 49% (95% CI=45–53%) and 33% (95% CI=23–42%), respectively. VE against influenza–associated intensive care unit (ICU) admission was 49% (95% CI=38–58%) and against in-hospital death was 48% (95% CI=31–62%).

**Conclusion:**

2024–2025 seasonal influenza vaccines provided protection against influenza–associated hospitalizations among adults with evidence of decreased VE ≥ 120 days after vaccination. VE point estimates were higher against influenza B than against influenza A and among immunocompetent versus immunocompromised adults. VE against influenza–associated ICU admission and in-hospital death were similar to that against hospitalization.

**Disclosures:**

Zachary Weber, PhD, MS, Centers for Disease Control and Prevention, Contract #200-2019-F-06819: Grant/Research Support Duck-Hye Yang, PhD, Centers for Disease Control and Prevention, Contract #200-2019-F-06819: Grant/Research Support Stephanie Irving, MHS, Westat: Grant/Research Support Sara Y. Tartof, PhD, MPH, Centers for Disease Control and Prevention: Grant/Research Support Nicola P. Klein, MD, PhD, AstraZeneca: Grant/Research Support|Centers for Disease Control and Prevention: Grant/Research Support|GlaxoSmithKline: Grant/Research Support|Janssen: Grant/Research Support|Merck: Grant/Research Support|Moderna: Grant/Research Support|Pfizer: Grant/Research Support|Sanofi Pasteur: Grant/Research Support|Seqirus: Grant/Research Support Shaun J. Grannis, MD, MS, Centers for Disease Control and Prevention: Grant/Research Support|National Institutes of Health NCATS: Grant/Research Support|National Institutes of Health NIMH: Grant/Research Support Toan Ong, PhD, Centers for Disease Control and Prevention via Westat: Grant/Research Support|Patent Title: Systems and Methods For Record Linkage: Patent Number: PCT/US2018/047961|PCORI: Travel Support|Regenstrief Institute: Advisor/Consultant|Regenstrief Institute: Travel Support Sarah W. Ball, MPH, ScD, Centers for Disease Control and Prevention, Contract #200-2019-F-06819: Grant/Research Support|Centers for Disease Control and Prevention, Contract #75D30121D12779: Grant/Research Support|Novavax: Grant/Research Support Malini B. DeSilva, MD, MPH, Centers for Disease Control and Prevention Vaccine Safety Datalink: Grant/Research Support|Westat: Grant/Research Support Padma Kppolu, MPH, Westat: Grant/Research Support S. Bianca Salas, MPH, Centers for Disease Control and Prevention: Grant/Research Support|Pfizer: Grant/Research Support Lina S. Sy, MPH, AstraZeneca: Grant/Research Support|Dynavax: Grant/Research Support|GlaxoSmithKline: Grant/Research Support|Moderna: Grant/Research Support Bruno Lewin, MD, Centers for Disease Control and Prevention: Grant/Research Support|National Institutes of Health: Grant/Research Support Richard Contreras, MS, Centers for Disease Control and Prevention: Grant/Research Support Ousseny Zerbo, PhD, Centers for Disease Control and Prevention: Grant/Research Support|Moderna: Grant/Research Support|National Institutes of Health: Grant/Research Support|Pfizer: Grant/Research Support John R. Hansen, MPH, Centers for Disease Control and Prevention: Grant/Research Support Lawrence Block, MPH, MPA, Centers for Disease Control and Prevention: Grant/Research Support Karen B. Jacobson, MD, MPH, Centers for Disease Control and Prevention: Grant/Research Support|National Institutes of Health: Grant/Research Support|Pfizer: Grant/Research Support William F. Fadel, PhD, Centers for Disease Control and Prevention: Grant/Research Support Catia Chavez, MPH, Westat: Grant/Research Support Adam Yates, PhD, Beehive Study: Grant/Research Support|Centers for Disease Control and Prevention, Contract #200-2019-F-06819: Grant/Research Support Lindsey Kirshner, MPH, Centers for Disease Control and Prevention, Contract #200-2019-F-06819: Grant/Research Support Charlene E. McEvoy, MD, MPH, Astra Zeneca: Grant/Research Support|Centers for Disease Control and Prevention: Grant/Research Support|Department of Defense: Grant/Research Support|GlaxoSmithKline: Grant/Research Support|National Institutes of Health: Grant/Research Support|PCORI: Grant/Research Support Karthik Natarajan, PhD, Centers for Disease Control and Prevention: Grant/Research Support

